# Tension Pneumoperitoneum: A Rare Complication of Cardiopulmonary Resuscitation (CPR)

**DOI:** 10.7759/cureus.60743

**Published:** 2024-05-21

**Authors:** M Samee, A Samee, Y Zubair, A Samee

**Affiliations:** 1 General Medicine, University Hospital of North Midlands, Stoke On Trent, GBR; 2 General and Colorectal Surgery, Royal Oldham Hospital, Manchester, GBR; 3 Medicine, Medical University, Plovdiv, BGR

**Keywords:** spontaneous pnemothorax, surgical emergencies, emergency medical service, cardiopulmonary resucitation, pneumo peritoneum

## Abstract

Tension pneumoperitoneum is a surgical emergency. Although rare, failure to diagnose and treat the condition may be lethal. Hence, being aware of this phenomenon, particularly in scenarios involving cardiopulmonary resuscitation (CPR), is important. Existing literature emphasises immediate abdominal needle decompression as the initial management followed by close monitoring and keeping a low threshold for surgical intervention as a definitive measure. We decided to write up this case report to raise awareness that a tension pneumoperitoneum can result as a complication of CPR, a well-known and widely practiced algorithm.

## Introduction

Pneumoperitoneum or the presence of free intraperitoneal gas is a well-known consequence of gastrointestinal visceral perforation, abdominal operation, or mechanical ventilation [1]. Tension pneumoperitoneum, on the other hand, is a rare complication in which the free intraperitoneal gas accumulates under pressure, resulting in hemodynamic and ventilatory compromise and thus necessitating urgent intervention.

We report a case of a 67-year-old female patient who developed a tension pneumoperitoneum during cardiopulmonary resuscitation (CPR) following a cardiac arrest. Abdominal examination revealed a significantly tense distended abdomen. Urgent imaging revealed free air under the diaphragm causing significant abdominal distension and compressing abdominal viscera. Immediate management via abdominal needle decompression resulted in a prompt return of cardiac output and spontaneous circulation. The patient was subsequently transferred to the intensive care unit (ICU) following resuscitation.

## Case presentation

An unwell 67-year-old lady was admitted to the Accident and Emergency (A&E) department and CPR was commenced. Her medical morbidities included type 2 diabetes and ischemic heart disease. On arrival, her temperature was 36.8 degrees, her pulse of 130, and her blood pressure was 80/60 millimetres of mercury. Her blood results were essentially within normal limits except for serum troponin levels which were raised to 52 ng/mL (Table 1).

**Table 1:** Blood results on admission WBC - white blood cells, CRP - C-reactive protein, GFR - estimated glomerular filtration rate, ALT - alanine aminotransferase, INR - international normalized ratio

Parameter	Values	Reference range
Hb	13.4	13-18 g/dL
WBC	5.7	4.0 -11.0 × 10^9^/L
Platelets	201	150-450 × 10^9^/L
CRP (serum)	<5	<0-9.9 mg/L
Urea (serum)	4.4	2.5-7.8 mmol/L
Sodium (serum)	136	133-146 mmol/L
Potassium (serum)	4.6	3.5-5.3 mmol/L
Creatinine (serum)	87	64-104 mmol/L
GFR	68	>60 mL/min/1.73m^2^
Albumin (serum)	40	35-50 g/L
Bilirubin (serum)	11	<21 µmol/L
Alkaline phosphatase (serum)	48	30-130 µmol/L
ALT (serum)	31	<55 µmol/L
INR	1	0.9-1.1
Prothrombin time	11.4	9.7-14.1s
Fibrinogen - derived	4.7	2.7-5.6 g/L
Troponin I (serum)	52	<0-34 ng/mL

The patient had pale mottled skin and urinary output was minimal. An urgent surgical opinion was sought when the resuscitating team noted a rapidly distending abdomen and minimum response to aggressive fluid resuscitation.

**Figure 1:**
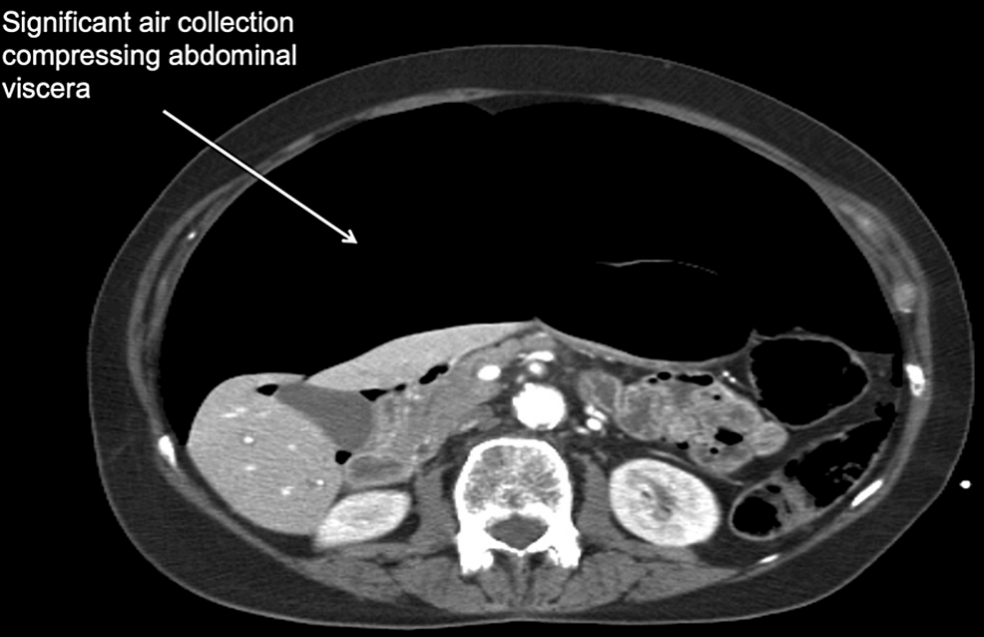
Axial CT scan image showing a significant collection of air compressing the abdominal viscera.

**Figure 2:**
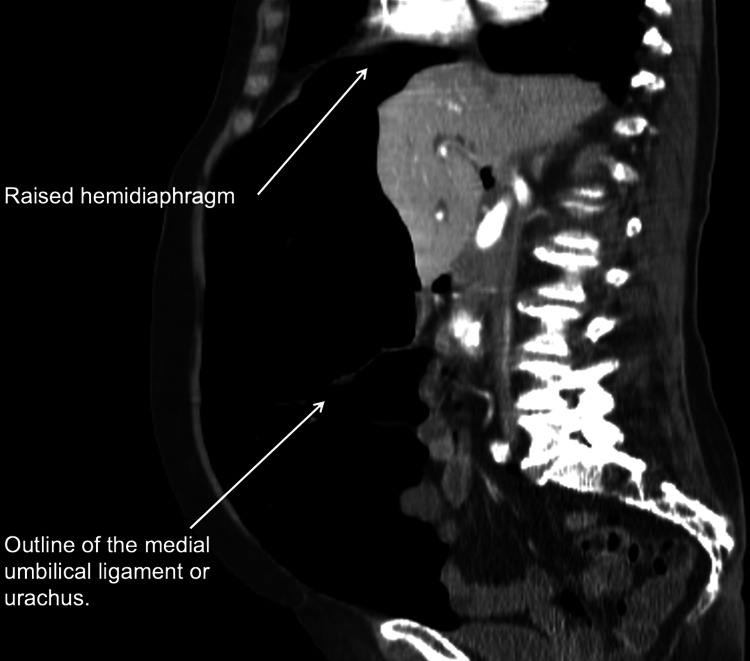
Sagittal CT scan image showing raised hemidiaphragm and the outline of the urachus. CT abdomen and pelvis with contrast: A massive pneumoperitoneum is seen, site of perforation could not be ascertained, no bowel wall thickening, and the other abdominal viscera are unremarkable.

On examination, the abdomen appeared to be quite significantly distended, tense, and tympanic throughout. Bowel sounds appeared faint but were present. Bilateral swelling in the groins was also noted. Assessment for signs of peritonism was not possible as the patient was intubated. The CT scan reported a significant pneumoperi toneum compressing the viscera. There was no free fluid. No findings to suggest visceral perforation were reported (Figures 1, 2).

An urgent abdominal decompression was carried out by inserting a needle in the umbilicus. A “significant hiss” was heard with air gushing out and, thus, softening of the abdomen. The resuscitation team reported an immediate improvement in the cardiac output. The patient was successfully transferred to the ICU for further management and was reviewed on a regular basis. Unfortunately, she suffered another cardiac arrest and died the next day.

## Discussion

Pneumoperitoneum usually indicates a perforated viscus and in the majority of cases will require emergency surgical intervention. Nonsurgical causes are seen in 10% of cases [1,2], and include mechanical ventilation [3], CPR [4,5], and pneumothorax [6]. One to three per cent of mechanically ventilated preterm babies develop nonsurgical pneumoperitoneum [7].

Tension pneumoperitoneum develops when the excess quantity of air gets trapped under pressure in the abdominal cavity causing abdominal tamponade [8]. Like tension pneumothorax, it is characterised by a "one-way" valve-like mechanism of air released into the abdominal cavity resulting in a progressive increase in intra-abdominal pressure [9]. This results in respiratory compromise due to a significantly raised hemi-diaphragm and a drop in cardiac output as a result of decreased venous return. It was first reported by Oberst [10], in 1917, in a case of grenade-induced gastric perforation where the liver was postulated to be lying over the perforation and acting as a one-way valve allowing the air to escape into but not out of the peritoneal cavity. Both spontaneous or iatrogenic bowel perforation (e.g., following endoscopy) may result in tension pneumoperitoneum and have been reported in the literature [11]. 

CPR, a widespread practice, is used for resuscitation in cardiac arrest involving chest compressions and rescue breaths [12]. Attempted CPR may result in rib fractures, sterna fractures, pneumothorax, cardiac, gastric contusion, and hepatosplenic injuries and is seen in 20%-65% of cases [12,13]. Gastric mucosal contusions and tearing [12,14] have been observed in about 10% of autopsy cases of unsuccessful CPRs [14].

The occurrence of a tension pneumoperitoneum, during CPR, leading to hemodynamic and ventilatory compromise, is a rare event. The exact aetiology is unknown. However, the mechanism is thought to be air escaping through natural diaphragmatic foramina into the abdominal cavity during CPR, where lungs are forcibly ventilated using positive pressure. A ball-valve effect allows one-way accumulation of the air into the abdominal cavity. The free air in the abdominal cavity creates a significantly raised intra-abdominal pressure, compressing the inferior vena cava, thus, compromising the venous return to the heart and a subsequent drop in cardiac output. It also splints up the diaphragm thus reducing lung compliance and causing respiratory compromise [1]. Studies in animals have shown that an elevation in intra-abdominal pressure results in decreased venous return to the heart resulting in reduced stroke volume, cardiac output, arterial PO2, and an increase in systemic vascular resistance [15,16]. Failure to recognise and decompress the tense abdomen urgently may result in respiratory and hemodynamic instability.

The radiological images will show significant gas under the diaphragm. The significantly distended abdomen will compress the abdominal viscera as seen in the CT scan image (Figure 1). The air may outline the ligaments or the urachus as a thin midline linear structure (Figure 2). The other radiological findings observed in tension pneumoperitoneum include a compressed superior vena cava, marked elevation of the diaphragm, and severely decreased thoracic volume [17].

The treatment for spontaneous tension pneumoperitoneum would include urgent percutaneous needle decompression of the abdominal cavity to relieve the rapidly rising intra-abdominal pressure. Radiological imaging may delay immediate intervention and ideally should be avoided [18]. However, it is a useful imaging modality to look out for other causes of pneumoperitoneum. The majority of these patients can be monitored closely without a need for an immediate laparotomy as resuscitation and optimisation are the priority. Close monitoring for signs of peritonitis is advised and surgical exploration may not be needed [19,20]. For tension pneumoperitoneum as a result of viscus perforation, emergency percutaneous decompression may not be a definitive treatment, only a method to bridge the time gap to definitive surgical repair.

Hemodynamic and respiratory compromise in addition to abdominal distension shortly after endoscopy are strongly suggestive of tension pneumoperitoneum due to iatrogenic bowel perforation. This is a rare but life-threatening condition, and it can be managed in a preclinical and clinical setting with emergency percutaneous needle decompression like tension pneumothorax followed by further management as guided by vital observation, clinical signs, and biochemical and radiological results.

## Conclusions

Spontaneous pneumoperitoneum has been reported in the literature and the management options include from close observation to surgical intervention. Tension pneumoperitoneum is defined as rapidly rising abdominal pressure (intra-abdominal hypertension) leading to a fall in cardiac output due to compromised venous return. Prompt recognition and intervention are key to a successful outcome as failure to recognise results is an inability to maintain cardiac output despite successfully treating other causes such as hypovolemia or pump failure. 

The attention toward the abdomen is often ignored during aggressive CPR. A tension pneumoperitoneum may develop during CPR. A rapidly progressing abdominal distension along with failure to sustain the cardiac output despite correcting hypovolemia should prompt the resuscitating team to look out for a distended abdomen as a possible contributor. Tension pneumoperitoneum leads to significant air entrapment in the abdominal cavity, thus, compromising venous return. It is a surgical emergency needing immediate intervention. As tension pneumothorax is managed by an urgent needle decompression, the management of tension pneumoperitoneum includes urgent percutaneous abdominal decompression using a needle. While the emergency is managed by urgent needle decompression, the possibility of intra-abdominal viscus injury or perforation should be kept in mind. Patients should be carefully monitored for signs of peritonitis. The treatment would be tailored according to individual cases based on close monitoring and repeated clinical, biochemical, and radiological assessment. 
